# Effects of Saccharomyces Cerevisiae Cultures on Performance and Immune Performance of Dairy Cows During Heat Stress

**DOI:** 10.3389/fvets.2022.851184

**Published:** 2022-03-01

**Authors:** Dewei Du, Lei Feng, Peng Chen, Wenbo Jiang, Yu Zhang, Wei Liu, Ruina Zhai, Zhiyong Hu

**Affiliations:** ^1^Ruminant Nutrition and Physiology Laboratory, College of Animal Science and Technology, Shandong Agricultural University, Taian, China; ^2^Beijing Enhalor International Tech Co., Ltd., Beijing, China; ^3^College of Animal Science, Xinjiang Agricultural University, Urumqi, China

**Keywords:** heat stress, Saccharomyces cerevisiae culture, mid-lactation dairy cows, milk yield, immunological performance, antioxidant capacity

## Abstract

The dairy farming industry is facing massive economic losses as heat stress continues to rise. The purpose of this study was to see how feeding Saccharomyces cerevisiae culture (SC) influences productive performance, lactation performance, serum biochemical indexes, hormonal level, antioxidant capacity, and immune function in mid-lactating cows during heat stress. Forty-five healthy mid-lactation dairy cows with comparable milk yield, lactation days, and parity were randomly divided into 3 groups (15 cows in each group). The control group (CON) was fed the basal diet, while the treatment groups were fed the basal diet + first Saccharomyces cerevisiae culture 100 g/d (SC-1) and the basal diet + second Saccharomyces cerevisiae culture 30 g/d (SC-2), respectively. The SC-1 and SC-2 groups with SC added in the treatment groups reduced rectal temperature and respiratory rate in heat-stressed cows (*P* < 0.05). The milk yield of SC-1 and SC-2 treatment groups was significantly higher than that of CON (*P* < 0.05). Except for somatic cell count, which was significantly lower in SC-1 and SC-2 than in CON (*P* < 0.05), there were no significant differences in the milk components. The addition of SC: (i) increased serum urea levels (*P* < 0.05), but there was no significant difference in glucose, total cholesterol, alanine transaminase, aspartate aminotransferase, total protein, albumin and alkaline phosphatase levels (*P* > 0.05); (ii) increased serum levels of immunoglobulin-A, immunoglobulin-G, immunoglobulin M, interleukin-4, interleukin-10 and heat shock protein-70 (*P* < 0.05), while decreasing serum levels of interleukin-1β, interleukin-6, interleukin-2, interferon-γ and tumor necrosis factor-α (*P* < 0.05); (iii) increased total antioxidant capacity, glutathione peroxidase and superoxide dismutase in serum (*P* < 0.05), while decreasing malondialdehyde; (iv) increased serum levels of glucocorticoids, insulin, cortisol and prolactin (*P* < 0.05), while decreasing the serum levels of triiodothyronine and thyroxine (*P* < 0.05). In conclusion, under the current experimental conditions, the addition of SC can reduce rectal temperature and respiratory rate in heat-stressed mid-lactation cows, reduce the number of somatic cells in milk and improve the mid-lactation cow performance. In addition, SC addition to the diet can raise serum urea levels, regulate serum hormone levels, boost antioxidant capacity in mid-lactation cows, and boost overall immunity.

## Introduction

Temperatures are rising in every region of the world as a result of global warming (1). According to NASA's Goddard Institute for Space Studies (GISS), global temperatures have risen at a rate of 0.15 to 0.20°C per decade since 1975 (2). As a result, extreme temperature events are becoming more common and severe (3). China has suffered from the effects of global warming in recent decades, which has exacerbated the occurrence of heat stress (4). Some data indicate that the temperature in China rose by an average of 1.4°C between 1951 and 2009 (5). China appears to be one of the regions most affected by high temperatures and one of the most severely heat-stressed (6).

Continued high heat stress can result in significant economic losses for the farming industry, particularly dairy farming (7). The temperature and humidity index (THI) is the sum of the effect of two variables, ambient temperature and humidity, is commonly used to assess the degree of heat stress in cows (8). The Holstein cow, which is known for its high productivity, is the most common breed of dairy cattle raised in China. However, it is extremely sensitive to temperature changes and will suffer heat stress if the ambient temperature exceeds 25°C and the THI exceeds 72 (9). Heat stress can cause a 35–40% reduction in milk production in cows (West, 2003), as well as affect the general health of the cow by reducing normal physiology, metabolism, hormones, antioxidant capacity and immune system (10). Furthermore, the increased incidence of mastitis in dairy cows during the summer poses a significant challenge to dairy farming enterprises (11).

Saccharomyces cerevisiae culture (SC) is a concentrated and dried product obtained by high-density liquid fermentation and deep solid fermentation with Saccharomyces cerevisiae as the strain; its composition includes yeast metabolites, yeast cells and denatured medium, which can significantly improve immunity, relieve stress and increase productivity (12). Supplementation of Saccharomyces cerevisiae culture in feed has been shown to improve milk production and reduce the inflammatory response of the organism in lactating cows (13). Moreover, we had demonstrated that Saccharomyces cerevisiae culture applied to heat-stressed sows could improve their lactation performance and antioxidant capacity (14). We discovered that the majority of previous studies in heat-stressed cows supplemented with Saccharomyces cerevisiae culture were concerned with effects on cow performance, reproductive capacity, lactation performance, and rumen fermentation parameters (15, 16). There was consideration given to the cow's immune system, antioxidant capacity, or hormone levels. It was previously unknown how Saccharomyces cerevisiae culture affects performance and cow health in mid-lactation cows subjected to heat stress via antioxidant capacity, immune capacity, and body hormone levels. Therefore, this study systematically investigated the effects of Saccharomyces cerevisiae culture on the production performance, serum biochemical indexes, hormone levels, antioxidant capacity, and immune indexes of dairy cows in the middle lactation under heat stress, and provided some data for the development and use of feed additives to mitigate heat stress in dairy cows.

## Materials and Methods

### Animals

The trial was conducted at the cattle farm of Xianghe Dairy Co. in Zaozhuang City, Shandong Province from July 2, 2021 to August 20, 2021. Forty-five Holstein cows in mid-lactation with similar milk yield (29.4 ± 3.7), parity (1.8 ± 0.6), days in lactation (158 ± 14), and good health were selected for the trial and divided into three groups of 15 cows each in a randomized complete block design. The control group (CON) was fed a basal diet, while the treatment groups were fed a basal diet + the first Saccharomyces cerevisiae culture at a rate of 100 g/d (SC-1) and a basal diet + the second Saccharomyces cerevisiae culture at a rate of 30 g/d (SC-2), respectively. Cows in the treatment groups were fed SC separately at morning feeding. The trial lasted 60 d, with a 10-d adaptation period and a 50-d formal trial period. The base ration is a Total Mixed Ration (TMR) formulated following the Dairy Cattle Feeding Standard (NY/T34-2004). Table 1 lists the ingredients and nutritional levels. The test cows were kept in loose pens with sprinklers and fans on to keep them during the test period, allowing them to feed and drink freely. The cows were fed three times a day (7:00, 13:00, 18:30) and milked three times a day (5:30, 12:30, 8:30). The Ethical Commission of Shandong Agricultural University reviewed and approved the animal study.

**Table 1:** Ration composition and nutrient levels (dry matter basis, %).

**Ingredients**	**Content**	**Nutrient levels**	**Contents**
Alfalfa hay	11.8	DM	95.9
Corn silage	47.0	CP	15.6
Syrup	3.1	EE	4.2
Mixed concentrates^a^	23.5	NDF	32
Fat powder	0.3	ADF	20.6
Flaked corn	7.8	Ca	0.64
Sugar beet pulp	2.6	P	0.44
Cottonseed	3.1		
Soybean meal	0.8		
Total	100.0		

a *The mixed concentrate contains self-contained concentrate from Xianghe Dairy Company and lactating cow concentrate supplement [8862], each kg of self-contained ingredients contains 0.55 kg of corn, 0.11 kg of DDGS, 0.07 kg of bran, 0.19 kg of soybean meal, 0.045 kg of Rovimix, 0.027 kg of baking soda, 0.01 kg of magnesium oxide and 0.0027 kg of calcium; The raw material composition of 8,862 includes corn, corn by-product, wheat by-product, cotton meal, soybean meal, soybean peel, sodium chloride, rock flour, feed grade calcium hydrogen phosphate, vitamin and mineral premix, etc*.

### Material

The first Saccharomyces cerevisiae culture “Baihuibang” and the second Saccharomyces cerevisiae culture “Baihuibang 4C” were provided by Beijing Enhalor International Tech Co., Ltd.

### Determination of Temperature and Humidity Index (THI)

An electronic temperature and humidity meter (purchased from Shandong Renke Measurement & Control Technology Co., Ltd. Model: COS-04) was used to record Temperature (°C) and relative humidity (%) at the same height as the cow's head during the experiment. Temperature (°C) and relative humidity (%) data were collected daily in the Dairy barn at 07:00, 14:00, and 22:00, and converted to temperature-humidity index (THI) (17). THI is calculated as follows:


$$\begin{array}{l} \text{THI}=\left(0.8\times \text{T}\right)+\left[\left(\text{RH}/100\right)\times \left(\text{T}-14.3\right)\right]+46.4 \end{array}$$

where T denotes the cowshed temperature (°C); RH denoted the relative humidity of the cowshed (%).

### Collection and Determination of Forage Samples

On day 50 of the formal experimental period using the quartering technique, forage samples were collected, dried in an oven at 65°C for 72 h, returned to moisture for 12 h, and then crushed to test the following: dry matter (DM) [GB 6435-86], crude protein (CP) [GB/T 6432-94], crude fat (EE) [GB/T 6433-1994], neutral detergent fiber (NDF), acid detergent fiber (ADF), calcium (Ca) [GB/T 6436-2002], phosphorus (P) [GB/T 6437-2002]. The content of neutral detergent fiber (NDF) and acid detergent fiber (ADF) was determined using the Van Soest method (18).

### Determination of Milk Yield and Milk Samples

The milk yield of each test cow was recorded on 2 consecutive days every 10 days during the formal experimental period. Data on milk production data were collected at 05:30, 12:30, and 20:00 each day, and then summarized into daily milk production. On day 50 of the official test period, milk samples were collected from three milking periods, mixed (50 mL) in a 4:3:3 ratio. Next, a potassium dichromate preservative was added, and the milk was sent to the Dairy Research Center of Shandong Academy of Agricultural Sciences for examination of milk fat percentage, milk protein percentage, somatic cell count, and urea nitrogen.

### Measurement of Blood Indicators

On the 50th day of the formal experimental period, blood was drawn from each cow at the caudal root vein before the morning feeding. After that, 20 mL of blood was drawn with a disposable syringe, coagulated with a procoagulation tube, and left for 30 min. Next, the blood was centrifuged for 5 min at 3,000 r/min, and the supernatant was divided into 1.5 mL centrifuge tubes and immediately stored in liquid nitrogen. Ten cows in each group were randomly selected to test blood parameters, including T3, T4, GC, IgA, IgG, IgM, PRL, HSP70, INS, COR, IL-1β, IL-6, IL-2, IL-4, IL-10, TNF-α, IFN-γ through enzyme-linked immunosorbent assay (ELISA) and ELISA kits with Angle gene products. Malondialdehyde (MDA) was determined using the MDA test kit, purchased from the Angle gene. The superoxide dismutase (SOD) kit, purchased from the Angle gene, was used to measure (SOD). Glutathione peroxidase (GSH-PX) was determined using the GSH-PX test kit, purchased from the Angle gene. Total antioxidant capacity (T-AOC) was determined using an Angle gene total antioxidant capacity (T-AOC) kit. A fully automated biochemical analyzer (Beckman Coulter Model: AU680) was used to determine serum biochemical parameters.

### Data Statistics

The test data were compiled using Excel 2010 software; the test data were subjected to Shapiro-Wilk normal distribution test using the shapiro.test() function of R software (version 4.1.1), chi-square test using the bartlett.test() function, and the ANOVA one-way ANOVA using the aov() function analysis of variance (ANOVA). The lsmeans package was used to compute SE values, and Duncan's method from the agricolae package was used for multiple comparisons. 0 < *P* < 0.001 represents a highly significant difference; 0.001 < *P* < 0.01 represents a very significant difference; 0.01 < *P* < 0.05 represents a significant difference; 0.05 < *P* < 0.1 represents a significant trend of difference. The images in the paper were created with the ggplot2 package of R software.

## Results

### Changes in Average Temperature and Humidity and Average Temperature and Humidity Index (THI) of the Dairy Barn During the Formal Experimental Period of 50 Days

**Figure 1:**
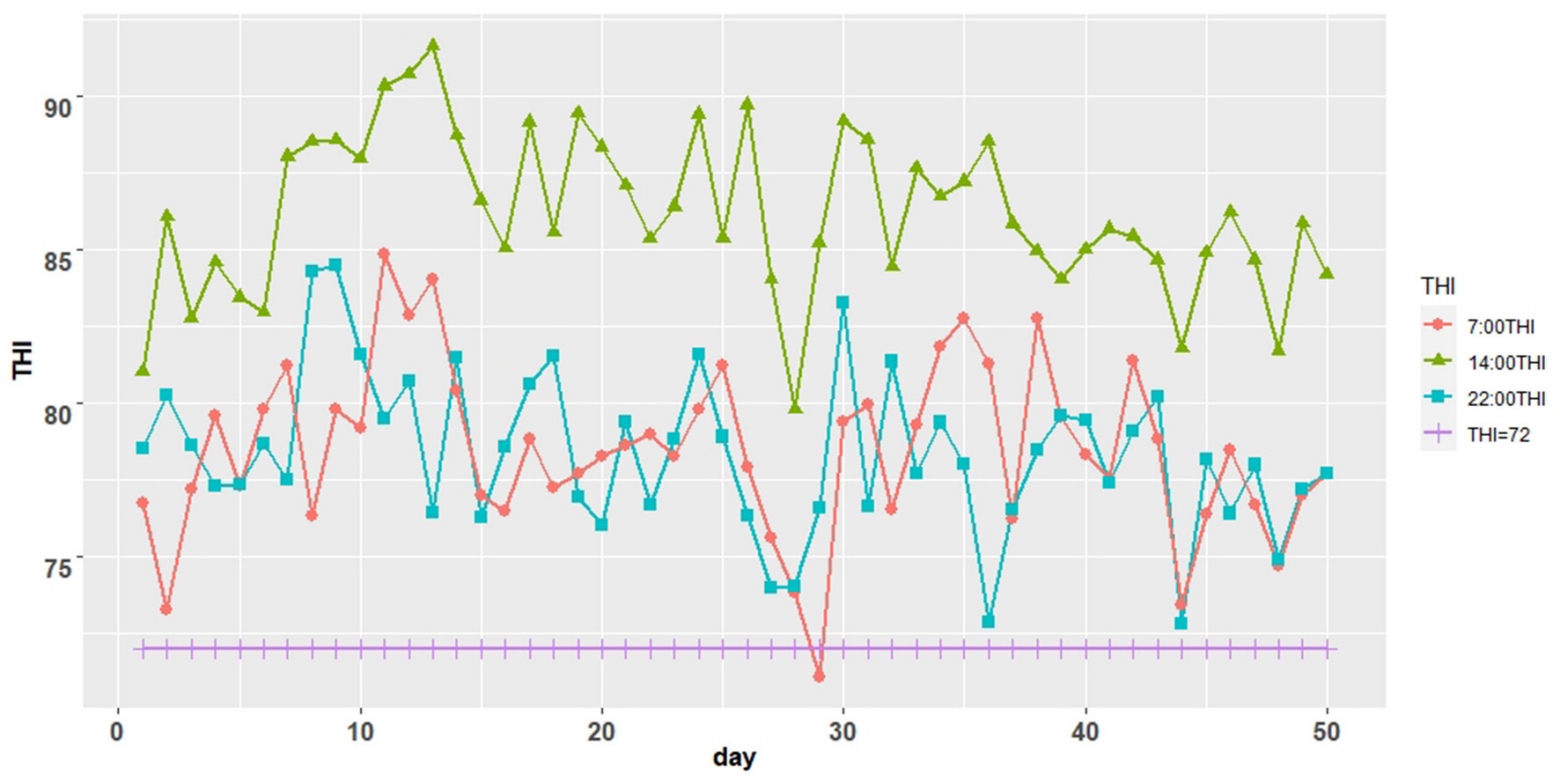
Trend graph of temperature and humidity index (THI) of Dairy barn.

The THI was >72 for almost all periods during the formal trial period (Figure 1). The average temperature of the Dairy barn during the formal trial period was a minimum of 26.9°C, higher than the temperature threshold of 25.8°C for the appropriate feeding environment for lactating cows (Table 2).

**Table 2:** Average temperature and humidity and average temperature and humidity index of the barn during the official test period (50 days).

**Time**	**Average**	**Average relative**	**Average THI**
	**temperature (**°**C)**	**humidity (%)**	
7:00	27.1	82.8	78.47
14:00	33.6	67.2	86.18
22:00	26.9	83.2	78.37

### Effect of Different Saccharomyces Cerevisiae Cultures on Rectal Temperature and Respiratory Rate in Dairy Cows

Rectal temperature and respiratory rate were significantly lower (*P* < 0.05) in the SC-1 and SC-2 groups of cows fed with Saccharomyces cerevisiae cultures than in the CON group; however, there was no significant difference between the SC-1 and SC-2 groups (Table 3).

**Table 3:** Effect of different Saccharomyces cerevisiae cultures on rectal temperature and respiratory rate in dairy cows.

**Items**	**Groups**			**SEM**	***P*-value**
	**CON**	**SC-1**	**SC-2**		
Rectal temperature/°C	38.89 ± 0.45^a^	38.58 ± 0.48^b^	38.69 ± 0.49^b^	0.06	0.0003
Respiratory rate/(bpm)	65.19 ± 10.05^a^	60.58 ± 13.1^b^	60.36 ± 12.44^b^	1.39	0.0216

*Data marked with the same lowercase letter indicate no significant difference (P > 0.05), different lowercase letters indicate significant difference (P <0.05). 0 < P <0.001 represents a highly significant difference; 0.001 < P <0.01 represents a very significant difference; 0.01 < P <0.05 represents a significant difference; 0.05 < P <0.1 represents a significant trend of difference. The same holds for the tables below*.

### Effect of Different Saccharomyces Cerevisiae Cultures on the Productive Performance of Dairy Cows

As shown in Table 4, the milk yield of SC-1 and SC-2 in the treatment group was significantly higher than that of CON (*P* < 0.05), and there was no significant difference between SC-1 and SC-2 (*P* > 0.05), but SC-1 had a higher milk yield than SC-2. The three groups did not differ significantly in terms of milk fat rate, milk protein rate, or urea nitrogen content (*P* > 0.05). The content of somatic cell count (SCC) was significantly lower in SC-1 and SC-2 than in CON (*P* < 0.05).

**Table 4:** Effect of different Saccharomyces cerevisiae cultures on the productive performance of dairy cows.

**Items**	**Groups**			**SEM**	***P*-value**
	**CON**	**SC-1**	**SC-2**		
Milk yield (kg/d)	26.35 ± 4.88^b^	28.87 ± 4.99^a^	28.72 ± 4.79^a^	0.40	<0.0001
Milk fat percentage/%	3.33 ± 1.57^a^	3.23 ± 1.37^a^	3.08 ± 1.41^a^	0.38	0.8940
Milk protein percentage/%	3.17 ± 0.15^a^	3.24 ± 0.27^a^	3.15 ± 0.15^a^	0.06	0.5000
Milk Urea Nitrogen (ng/dL)	10.9 ± 3.01^a^	10.90 ± 3.40^a^	10.40 ± 3.95^a^	0.99	0.9260
SCC/(10 k/mL)	8.14 ± 9.40^a^	2.86 ± 2.07^b^	3.29 ± 3.99^b^	1.61	0.0461

*Different letters (a, b) represent significant differences*.

### Effect of Different Saccharomyces Cerevisiae Cultures on Serum Biochemical Indices of Dairy Cows

As depicted in Table 5, the urea content was significantly higher in the SC-1 and SC-2 groups than in the CON group (*P* < 0.05), but there was no significant difference in the content of glucose, cholesterol, Alanine transaminase, Aspartate aminotransferase, total protein, albumin, and alkaline phosphatase (*P* > 0.05).

**Table 5:** Effect of different Saccharomyces cerevisiae cultures on serum biochemical indices of dairy cows.

**Items**	**Groups**			**SEM**	***P*-value**
	**CON**	**SC-1**	**SC-2**		
GLU (mmol/L)	1.99 ± 0.59^a^	1.71 ± 0.48^a^	1.74 ± 0.57^a^	0.18	0.4970
TC (mmol/L)	5.71 ± 0.75^a^	6.14 ± 1.73^a^	5.39 ± 0.81^a^	0.37	0.3850
ALT (U/L)	34.6 ± 6.35^a^	39.9 ± 8.51^a^	36.6 ± 7.14^a^	2.34	0.3740
AST (U/L)	84.6 ± 21.02^a^	87.4 ± 16.39^a^	91.6 ± 20.13^a^	6.40	0.7540
TP (g/L)	83.43 ± 7.43^a^	83.94 ± 6.53^a^	82.23 ± 4.78^a^	2.01	0.8270
ALB (g/L)	25 ± 1.58^a^	26.7 ± 2.51^a^	26.4 ± 1.65^a^	0.62	0.1410
UREA (mmol/L)	5.29 ± 0.51^a^	5.46 ± 0.51^b^	6.08 ± 0.84^b^	0.20	0.0 248
AKP (U/L)	53.2 ± 18.43^a^	60.9 ± 14.31^a^	50.4 ± 11.84^a^	4.88	0.3180

*Different letters (a, b) represent significant differences*.

### Effect of Different Saccharomyces Cerevisiae Cultures on Serum Hormone Indices in Dairy Cows

As shown in Table 6, the levels of glucocorticoids, insulin, cortisol, and prolactin were significantly higher in the SC-1 and SC-2 groups than in the CON group (*P* < 0.05), while the levels of triiodothyronine and thyroxine were significantly lower in the SC-1 and SC-2 groups (*P* < 0.05). The levels of glucocorticoids, insulin, and cortisol were significantly higher in the SC-1 group than in the SC-2 group, while the levels of triiodothyronine and thyroxine content in the SC-1 group were significantly lower.

**Table 6:** Effect of different Saccharomyces cerevisiae cultures on serum hormone indices in dairy cows.

**Items**	**Groups**			**SEM**	***P*-value**
	**CON**	**TestA**	**TestB**		
GC (ng/mL)	23.91 ± 0.75^c^	33.07 ± 0.89^a^	28.06 ± 0.81^b^	0.26	<0.0001
INS (mU/mL)	8.91 ± 0.43^c^	12.45 ± 0.53^a^	10.82 ± 0.52^b^	0.16	<0.0001
COR (ng/mL)	70.57 ± 3.41^c^	100.58 ± 5.43^a^	85.28 ± 5.56^b^	1.55	<0.0001
T3 (ng/mL)	1.94 ± 0.07^a^	1.19 ± 0.04^c^	1.52 ± 0.05^b^	0.02	<0.0001
T4 (ng/mL)	90.09 ± 3.49^a^	66.08 ± 3.74^c^	78.12 ± 2.77^b^	1.06	<0.0001
PRL (μIU/mL)	2,684.84 ± 158.45^b^	2,999.43 ± 153.59^a^	2,900.27 ± 129.5^a^	46.70	0.0002

*Different letters (a–c) represent significant differences*.

### Effect of Different Saccharomyces Cerevisiae Cultures on Serum Antioxidant Indices in Dairy Cows

Table 7 shows that the total antioxidant capacity, glutathione peroxidase, and superoxide dismutase contents were significantly higher in the SC-1 and SC-2 groups than in the CON group (*P* < 0.05), with the total antioxidant capacity and superoxide dismutase contents in the SC-1 group significantly higher than in the SC-2 group. Malondialdehyde levels were significantly lower in the SC-1 and SC-2 groups than in the CON group (*P* < 0.05), with the SC-1 group having a significantly lower malondialdehyde level than the SC-2 group.

**Table 7:** Effect of different Saccharomyces cerevisiae cultures on serum antioxidant indices in dairy cows.

**Items**	**Groups**			**SEM**	***P*-value**
	**CON**	**SC-1**	**SC-2**		
T-AOC (mmol/L)	0.16 ± 0.01^c^	0.23 ± 0.01^a^	0.19 ± 0.01^b^	0.00	<0.0001
GSH-PX (U/mL)	12.01 ± 1.08^b^	13.53 ± 0.63^a^	12.83 ± 0.81^a^	0.27	0.0020
MDA (nmol/mL)	6.63 ± 0.31^a^	4.44 ± 0.22^c^	5.32 ± 0.28^b^	0.09	<0.0001
SOD (U/mL)	69.38 ± 1.73^c^	77.84 ± 1.79^a^	73.89 ± 2.17^b^	0.60	<0.0001

*Different letters (a–c) represent significant differences*.

### Effect of Different Saccharomyces Cerevisiae Cultures on Serum Immune Indices in Dairy Cows

The levels of IgA, IgG, IgM, IL-1β, IL-6, IL-2, IL-4, IL-10, TNF-α, IFN-γ, and HSP70 in serum were significantly different in the SC-1 and SC-2 groups compared to the CON group (*P* < 0.05), as shown in Table 8. The contents of IL-10 and HSP70 were significantly higher in SC-1 and SC-2 groups than in the CON group, while the contents of IL-1β, IL-6, IL-2, IFN-γ, and TNF-α were significantly lower in the CON group. The contents of IgA, IgM, IL-10, and HSP70 in the SC-1 group were significantly higher than those in the SC-2 group, while the contents of IL-1β, IL-6, IFN-γ, and TNF-α in the SC-2 group were significantly were lower.

**Table 8:** Effect of different Saccharomyces cerevisiae cultures on serum immune indices in dairy cows.

**Items**	**Groups**			**SEM**	***P*-value**
	**CON**	**SC-1**	**SC-2**		
IgA (μg/mL)	20.02 ± 0.71^c^	25.75 ± 0.98^a^	22.87 ± 0.81^b^	0.27	<0.0001
IgG (μg/mL)	232.59 ± 13.78^b^	255.24 ± 11.59^a^	246.84 ± 12.74^a^	4.03	0.0028
IgM (μg/mL)	16.09 ± 0.99^c^	20.06 ± 0.97^a^	18.24 ± 1.15^b^	0.33	<0.0001
IL-1β (pg/mL)	191.73 ± 8.16^a^	151.52 ± 7.87^c^	171.31 ± 7.14^b^	2.45	<0.0001
IL-6 (pg/mL)	255.71 ± 7.22^a^	221.54 ± 6.76^c^	236.15 ± 6.11^b^	2.12	<0.0001
IL-2 (pg/mL)	21.11 ± 0.67^a^	17.04 ± 0.56^c^	19.08 ± 0.54^b^	0.19	<0.0001
IL-4 (pg/mL)	412.39 ± 25.17^b^	446.26 ± 20.40^a^	433.77 ± 20.21^a^	6.97	0.0068
IL-10 (pg/mL)	359.46 ± 17.1^c^	401.21 ± 19.95^a^	378.06 ± 18.71^b^	5.89	0.0001
TNF-α (ng/L)	155.67 ± 7.36^a^	130.11 ± 6.61^c^	143.11 ± 6.78^b^	2.19	<0.0001
IFN-γ (pg/mL)	701.96 ± 34.36^a^	593.17 ± 27.01^c^	641.29 ± 32.08^b^	9.90	<0.0001
HSP70 (ng/mL)	7.8 ± 0.37^c^	10.34 ± 0.45^a^	9.13 ± 0.51^b^	0.14	<0.0001

*Different letters (a–c) represent significant differences*.

## Discussion

### Effect of Adding Different Types of Saccharomyces Cerevisiae Cultures on Rectal Temperature and Respiratory Rate in Heat-Stressed Cows

Heat stress can have a significant impact on the development of our dairy industry. THI=72 has been widely adopted as the threshold value for the occurrence of heat stress in dairy cows over the last decade of heat stress research (19). In this trial, the THI was above 72 for almost all periods, and the times when it was below 72 were due to a brief cooling in the Zaozhuang area of Shandong Province because of the impact of Typhoon No. 6 “Fireworks” in China in 2021. So we believe the cattle were in a heat stress environment. During heat stress, the most common physiological indicators used to assess heat stress in cows are respiratory rate (RR) and rectal temperature (RT) (20). Animals dissipate excess heat from their bodies by increasing their respiratory rate (RR) and allowing it to evaporate in the environment (21). Some researchers, on the other hand, proposed that rectal temperature (RT) is a sensitive indicator of heat stress (22) and that small changes in RT can profoundly influence the function of tissues, organs, and endocrine and that such changes may reduce lactation and causing a variety of growth problems (23). Other studies suggest that as THI increases, so do RT and RR (24). In the present study, the addition of Saccharomyces cerevisiae cultures decreased ectal temperature and respiratory rate in cows during heat stress, possibly improving the adaptation of cows to heat stress. Also, we found the results of the same trial in a previous report by Shan and colleagues, where feeding chromium-rich yeast reduced rectal temperature and respiratory rate in lactating cows during heat stress (25).

### Effect of Adding Different Types of Saccharomyces Cerevisiae Cultures on the Performance of Heat-Stressed Cows

Heat stress causes a decrease in milk production as well as changes in milk composition (26, 27). In a previous study, lactating cows showed a linear decrease in milk production as THI increased from 60 to 80, with milk production decreasing by 0.13 kg/d for every 1 unit increase in THI (28), and this situation was more prevalent in high yielding cows (29). Additional evidence shows that the number and secretory activity of mammary epithelial cells in cows determine milk production (30). The presence of an intact mammary epithelial barrier, which is an indicator of good mammary gland function, is a major prerequisite for maintaining a high level of milk production (31). According to a study by Collier and colleagues, *in vitro* culture of bovine mammary epithelial cells at high ambient temperatures impaired cell proliferation and induced apoptosis (32), which could reduce milk production. In the present study, the addition of yeast additive improved milk production in cows during heat stress, possibly improving the adaptation of mammary epithelial cells to heat stress. This finding is consistent with the results of many other researchers, including Bruno and the team, who found that adding Saccharomyces cerevisiae cultures to the ration improved lactation performance of heat-stressed cows, increasing milk production from 42.2 to 43.4 kg/d. One study found an increase in somatic cell count (SCC) in milk from heat-stressed cows (33). This could be due to the phenomenon that heat stress reduces productive performance and immunity in cows via biochemical and biological pathways, increasing the incidence of intra-mammary infection (IMI), which leads to higher somatic cell counts (SCC) (34). In the present research, the addition of Saccharomyces cerevisiae cultures could reduce the level of SCC in milk. Furthermore, SCC is a predictor of IMI and also an indicator of milk quality and assessment of the degree of mastitis (35); as such, adding SC may reduce intra-dairy infection in cows during heat stress, improve milk quality, reduce the prevalence of mastitis, and play a positive role cow health during heat stress.

### Effect of Adding Different Types of Saccharomyces Cerevisiae Cultures on Serum Biochemical Indices of Heat-Stressed Cows

Urea is the end product of ammonia and amino acid metabolism in animals; some urea will re-enter the metabolic process if the amount synthesized by the liver exceeds the amount of urea excreted in the urine (36). Cows can suffer from loss of appetite and reduced food intake during heat stress, which potentially results in a negative energy balance (37, 38). Evidence shows that this phenomenon can cause high lipid mobilization from body fat reserves, lipid deposition in the liver, ketogenesis, and hypoglycemia (39). It has been demonstrated that serum urea concentrations are lower in cows with high lipidation and ketogenesis in early lactation when compared to healthy cows, which may be ascribed to a reduction in dry matter intake in relation to hepatic metabolism (40). In the present trial, the addition of Saccharomyces cerevisiae cultures potentially increased urea levels, which may have improved the negative energy balance of cows and increased liver metabolism. There were no significant differences in other serum biochemical indicators. Niacin (NA), an important vitamin in vitamin B that elicits a vasodilatory response, is beneficial for heat-stressed cows. In a study by Khan and colleagues, the addition of NA increased plasma urea concentrations in crossbred cows under heat stress (41), which is consistent with our trials. Previously, it was demonstrated that Saccharomyces cerevisiae culture strains are pro-nutrients of all group B vitamins and have high levels of vitamin B (42). Therefore, the elevated urea in the present trial following the addition of Saccharomyces cerevisiae cultures may be linked to group B vitamins and positively influence liver metabolism.

### Effect of Adding Different Types of Saccharomyces Cerevisiae Cultures on Serum Hormones, Antioxidants, and Immune Indices in Heat-Stressed Cows

Heat stress can cause a variety of physiological and hormonal responses via various molecular mechanisms (43). According to one study, cortisol concentrations in cow blood increased after prolonged heat stress exposure (44), which is thought to be one of the main adaptive mechanisms by which the organism is negatively influenced (45). Munksgaard and colleagues also noted that increased cortisol levels ensured nutrient mobilization and availability in response to the stressor (46). In the present trial, cortisol levels were significantly higher in cows fed Saccharomyces cerevisiae cultures, which could mobilize nutrients and ensure the supply of substances in the body. Compelling evidence shows that increased milk production necessitates higher levels of glucocorticoids to provide enough energy for galactose production. Cortisol, the main glucocorticoid in galactose production, not only enhances the role of prolactin (PRL) in stimulating mammary epithelial cell differentiation but also increases the expression of milk protein genes during lactation (47). These data imply that prolactin and cortisol can promote increased milk production in cows. Herein, we found that as the concentration of cortisol in the test group increased, so did the level of prolactin, which, when combined with the fact that the combined milk yield increased, suggests that adding Saccharomyces cerevisiae cultures can cause cortisol and prolactin to act synergistically in cows to improve their productivity. Heat stress is well known to cause an increase in insulin concentrations (48). Multiple pieces of evidence indicate that insulin action enables the body to respond effectively to heat stress and minimizes heat stress-induced injury (49, 50), and the increased serum insulin concentrations in cows supplemented with SC in our experiment may be because the addition of SC promotes insulin production in the body, successively reducing heat stress-induced damage in cows.

HSP70 is a key gene in the heat shock protein (HSP) family that has been linked to heat stress response (51). The structure of HSP70 is highly conserved, and it may be involved in protein folding, assembly, and degradation. HSP70 synthesis in cells is low under normal conditions. Under stress, however, they are rapidly synthesized and transferred to the nucleus, nucleolus, and other regions to improve the heat resistance of the organism and play a protective role (52). This heat resistance is positively correlated with the level of HSP70 expression (53). This allows the organism to adapt to heat stress earlier, and this protects tissues and organs from heat stress damage faster and more effectively, as well as improves the organism's resistance to stress (54). Herein, there was a significant increase in HSP70 levels in the treatment group, most likely because the addition of Saccharomyces cerevisiae cultures could stimulate the rapid HSP70 synthesis in cows. Notably, Rhoads et al. (50) revealed that HSP70 may improve insulin function and that modulating the insulin-HSP axis during heat stress can improve animal health and productivity, and the treatment group did show a significant increase in insulin metrics as well as a positive correlation between insulin levels and HSP70 levels, which was consistent with previous findings. However, we saw the opposite conclusion in a study where the addition of Saccharomyces cerevisiae cultures to periparturient cows under heat stress reduced plasma levels of HSP70 (55), and we speculate that this may be due to the different periods of the herd. Thyroid hormone (T3) is the primary hormone secreted by the thyroid gland, and triiodothyronine (T4) is a deiodination product of T3 and is only biologically active when deiodinated (56). Because the thyroid gland regulates basal metabolic rate in lactating cows, increased release of T4 and T3 increases basal metabolic rate and body heat production (57). Under hot climatic conditions, the decrease in thyroid hormone concentration can be viewed as an adaptive mechanism to reduce metabolic heat production (58). Reduced food intake during heat stress has been linked to thyroid activity suppression, which consequently decreases thyroid hormone levels (59). According to our findings, the addition of yeast additive reduced serum levels of T3 and T4 in heat-stressed cows as well as the metabolic heat production of the organism for better adaptation to the hot environment. The same findings were reported previously (60).

The immune system is one of the mechanisms for resisting stress caused by environmental changes. Heat stress can reduce dry matter intake in cattle, thereby negatively influencing nutrient absorption, and, as a result, the immune system and inflammatory response (61). Serum immunoglobulin is a marker of humoral immunity that promotes monocyte and macrophage phagocytosis and can bind to antigens to produce multiple biological effects. Immunoglobulin-G (IgG) is the major immunoglobulin that promotes the phagocytosis of pathogens and neutralizes bacterial toxins by immune cells. High IgG concentrations in dairy cow blood are thought to boost immunity. Immunoglobulin-A (IgA) is found in low concentrations in serum but provides significant defense against invading pathogens. Immunoglobulin-M(IgM) lyses bacteria and blood cells and neutralizes viruses, but its retention time is shorter (62, 63). Some studies have demonstrated that heat stress suppresses biological immune functions. One of these effects is a decrease in serum immunoglobulin levels (64). In the present experiment, feeding Saccharomyces cerevisiae cultures significantly increased serum levels of IgG, IgA, and IgM in heat-stressed cows, which improved the immunity of the cow organism and reduced heat stress-induced damage to the immune system. As a result, the ability of the organism to resist heat stress is improved.

CD4+ T cells are immune cells that are classified as T helper cells in the lymphocyte classification. CD4+ T cells are classified as either helper T cell 1 (Th1) or helper T cell 2 (Th2) cells. Their presence or activation is thought to have a regulatory effect on immune behavior. Recent studies have shown that the main mechanism for maintaining or restoring homeostasis in the diseased immune system is the relative increase in Th2 cell activity to the relative suppression of Th1 cells (65). Th1 cells mainly secrete IL-2 and IFN-γ, whereas th2 cells mainly secrete IL-4 and IL-10 (66). Th1 and Th2 cells, respectively, regulate cellular and humoral immunity. Th1 cells promote the inflammatory response, while Th2 cells promote the anti-inflammatory response (67). Researchers have suggested that high temperature is associated with the downregulation of Th1 cytokines and upregulation of Th2 cytokines, thereby suppressing cellular immunity (68, 69). In our study, we found that the levels of IL-2 and IFN-γ decreased, while the levels of IL-4 and IL-10 increased, which is consistent with previous findings; these data imply that yeast additions potentially reduced inflammation in heat-stressed cows and improved the anti-inflammatory response of the organism. Intriguingly, it has been proposed that glucocorticoids may suppress cellular immunity, resulting in a preferential shift toward th2-mediated humoral immunity (70), which is consistent with our findings. Heat stress has also been shown to increase cortisol concentration in the blood and to inhibit the production of cytokines such as interleukin-4 (IL-4), IL-6, interferon (IFN), and tumor necrosis factor- (TNF-) (71). Elsewhere, a study demonstrated that TNF-α and IL-1β directly promote inflammatory responses in cows, while IL-10 mediates anti-inflammatory responses in cows (72), and that interleukin (IL-6) is a known inflammatory cytokine that promotes inflammation in the organism (73). Herein, elevated serum cortisol concentrations were reported in the treatment group, whereas the levels of inflammatory factors such as TNF-α, IL-6, and IL-1β decreased. These results suggest that the addition of Saccharomyces cerevisiae cultures may regulate the immune system by influencing cortisol levels *in vivo*, and this would result in less inflammation in heat-stressed cows.

Heat stress disrupts the normal regulation of oxidants/antioxidants, causing severe cell damage via enzymatic and non-enzymatic activities (74). High temperatures can promote the occurrence of oxidative stress, which may be attributed to the excessive production of oxygen radicals by the organism and reduced antioxidant defenses (75). Increased oxygen free radicals result in the formation of malondialdehyde (MDA), which potentially induces cell death by damaging the DNA (76). Superoxide dismutase (SOD) is an enzyme that protects the organism and cells from damage caused by superoxide anion radicals and their active products. Glutathione peroxidase (GSH-PX) is a key enzyme in the antioxidant defense system of organisms, thought to protect various organisms from oxidative stress (77). Total antioxidant capacity (T-AOC), which represents total antioxidants in blood and body fluids, reflects the body's compensatory capacity to resist external stimuli (78). Malondialdehyde, a by-product of lipid peroxidation, is the most common indicator of lipid peroxidation and is regarded as a reliable method for determining the extent of lipid oxidative damage in cell membranes (79). In general, cows with relatively high levels of T-AOC, SOD, and GSH-Px and relatively low MDA values have good antioxidant capacity. In the present investigation, the levels of total antioxidant power, glutathione peroxidase, and superoxide dismutase increased in the experimental group, while malondialdehyde decreased, indicating that brewer's yeast culture can increase the antioxidant capacity of the cow organism under heat stress, reduce cell damage, and decrease the production of oxygen radicals. Harmon and colleagues reported a decrease in plasma antioxidant activity in heat-stressed Holstein cows in mid-lactation, which can indirectly verify our findings (80). In another investigation, researchers reported a 2-fold increase in malondialdehyde in skeletal muscle of acutely heat-stressed broiler chickens (81).

## Conclusion

The addition of Saccharomyces cerevisiae cultures to heat-stressed mid-lactation cows increases milk production and reduces the incidence of mastitis, but exerts no significant effect on milk composition. Moreover, the addition of Saccharomyces cerevisiae cultures improves the antioxidant capacity of the cows and can reduce inflammatory factors in the body, improve immunity, and synergize hormones in the body to alleviate the negative effects of heat stress. Thus, supplementing mid-lactation cows with Saccharomyces cerevisiae cultures can improve the antioxidant capacity and immunity level of the body under heat stress, as well as improve milk yield and cow performance to ensure healthy cow production. The experimental results demonstrated that the effect of Saccharomyces cerevisiae cultures in the SC-1 group is superior to that in the SC-2 group.

## Data Availability Statement

The original contributions presented in the study are included in the article/supplementary material, further inquiries can be directed to the corresponding authors.

## Ethics Statement

The animal study was reviewed and approved by Ethical Commission of Shandong Agricultural University.

## Author Contributions

DD, ZH, PC, and RZ conceived the project and designed the protocol. DD and WJ performed the experiments. DD, LF, YZ, and WL wrote the manuscript. All authors read and approved the final manuscript.

## Funding

This research was funded by the National Natural Science Foundation of China, grant number 32172760.

## Conflict of Interest

PC was employed by Beijing Enhalor International Tech Co., Ltd. The remaining authors declare that the research was conducted in the absence of any commercial or financial relationships that could be construed as a potential conflict of interest.

## Publisher's Note

All claims expressed in this article are solely those of the authors and do not necessarily represent those of their affiliated organizations, or those of the publisher, the editors and the reviewers. Any product that may be evaluated in this article, or claim that may be made by its manufacturer, is not guaranteed or endorsed by the publisher.
